# Newborn Screening for High-Risk Congenital Heart Disease by Dried Blood Spot Biomarker Analysis

**DOI:** 10.1001/jamanetworkopen.2024.18097

**Published:** 2024-06-24

**Authors:** Henning Clausen, Elin Friberg, Katarina Lannering, Aki Koivu, Mikko Sairanen, Mats Mellander, Petru Liuba

**Affiliations:** 1 Medical Faculty, Lund University, Sweden; 2 Children’s Heart Centre, Skane’s University Hospital, Lund, Sweden; 3 Medical Faculty, Gothenburg University, Gothenburg, Sweden; 4 Queen Silvia Children’s Hospital, Sahlgrenska University Hospital, Gothenburg, Sweden; 5 Revvity, Diagnostics Research & Development, Turku, Finland

## Abstract

**Importance:**

Congenital heart disease (CHD) is the most common human organ malformation, affecting approximately 1 of 125 newborns globally.

**Objectives:**

Assessing the performance of 2 diagnostic tests using minimal amounts of dried blood spots (DBS) to identify high-risk CHD compared with controls in a Swedish cohort of neonates.

**Design, Setting, and Participants:**

This diagnostic study took place in Sweden between 2019 and 2023 and enrolled full-term babies born between 2005 and 2023. All cases were identified through centralized pediatric cardiothoracic surgical services in Lund and Gothenburg, Sweden. Controls were followed up for 1 year to ensure no late presentations of high-risk CHD occurred. Cases were verified through surgical records and echocardiography.

**Exposure:**

High-risk CHD, defined as cases requiring cardiac surgical management during infancy due to evolving signs of heart failure or types in which the postnatal circulation depends on patency of the arterial duct. Using 3-μL DBS samples, automated quantitative tests for NT-proBNP and interleukin 1 receptor-like 1 (IL-1 RL1; formerly known as soluble ST2) were compared against established CHD screening methods.

**Main Outcomes and Measures:**

Performance of DBS tests to detect high-risk CHD using receiver operating characteristic curves; Bland-Altman and Pearson correlation analyses to compare IL-1 RL1 DBS with plasma blood levels.

**Results:**

A total of 313 newborns were included (mean [SD] gestational age, 39.4 [1.3] weeks; 181 [57.8%] male). Mean (SD) birthweight was 3495 (483) grams. Analyzed DBS samples included 217 CHD cases and 96 controls. Among the CHD cases, 188 participants (89.3%) were high-risk types, of which 73 (38.8%) were suspected prenatally. Of the 188 high-risk cases, 94 (50.0%) passed pulse oximetry screening and 36 (19.1%) were initially discharged after birth without diagnoses. Combining NT-proBNP and IL-1 RL1 tests performed well in comparison with existing screening methods and enabled additional identification of asymptomatic babies with receiver operating characteristic area under the curve 0.95 (95% CI, 0.93-0.98).

**Conclusions and relevance:**

In this diagnostic study, NT-proBNP and IL-1 RL1 DBS assays identified high-risk CHD in a timely manner, including in asymptomatic newborns, and improved overall screening performance in this cohort from Sweden. Prospective evaluation of this novel approach is warranted.

## Key Points

**Question:**

Can 2 circulating blood biomarkers—amino-terminal prohormone of brain natriuretic peptide (NT-proBNP) and interleukin 1 receptor-like 1 (IL-1 RL1)—be quantified in newborns using minimal amounts of dried blood spot (DBS) samples with reliable results for the early detection of congenital heart disease (CHD) serious enough to require cardiac surgery during infancy?

**Findings:**

In this diagnostic study of 313 newborns from Sweden, NT-proBNP and IL-1 RL1 were analyzed using 3 μL of DBS and discriminated controls from CHD cases well, including asymptomatic cases not identified by fetal ultrasound, postnatal clinical examination, or pulse oximetry.

**Meaning:**

These results suggest that novel diagnostic DBS tests performed well in comparison with existing screening methods for CHD, warranting prospective evaluation to improve early diagnosis of even asymptomatic cases.

## Teaser

This diagnostic study of individuals in Sweden born between 2005 and 2023 assesses the performance of blood biomarker testing for early detection of high-risk congenital heart disease.

## Introduction

Congenital heart disease (CHD) is the most common organ malformation in humans, affecting approximately 1 of 125 newborns.^1^ Critical CHD, defined as cases dependent on patency of the arterial duct for survival after birth, occurs in approximately 1 of 500 newborns and requires urgent medical attention to prevent circulatory collapse and early surgical management to stabilize the circulation.^2,3,4^ Such critical cases, along with other severe types of CHD that require cardiac surgery during infancy to prevent heart failure and mortality, can be considered high-risk CHD. There is a global need to improve early diagnosis of such high-risk cases while overcoming inequalities in CHD care provision to achieve optimal outcomes.^4^ Identifying such cases can be partially achieved by prenatal maternal ultrasound screening with detection in approximately half of cases, which in turn provides time for parental counselling, planning, and postnatal stabilization prior to surgery, even though these cases may often represent the most complex CHD forms.^5,6,7^ The remainder of cases will rely on timely, postnatal diagnoses to minimize morbidity and mortality using clinical examinations or additional newborn pulse oximetry screening, although the latter continues to have modest sensitivity with diagnoses, such as aortic coarctation, that represent significant challenges.^8,9^ We set out to develop quantitative DBS assays for the amino-terminal prohormone of brain natriuretic peptide (NT-proBNP) and interleukin 1 receptor-like 1 protein (IL-1 RL1; formerly known as soluble ST2) and evaluated these among a Swedish cohort of newborns. Biomarkers were selected based on published scientific data that have suggested a strong relationship between heart failure and elevated blood biomarker levels in various age groups and types of cardiac diseases, both in adults and children.^10,11,12,13,14,15,16^ To address the need for timely and efficient detection of high-risk CHD in newborns, we hypothesized that the cardiovascular biomarkers NT-proBNP and IL-1 RL1 could aid this process if analyzed using minimal amounts of dried blood spot (DBS) samples, as commonly used in newborn screening programs.

## Methods

This diagnostic study was performed according to the ethical principles of the Declaration of Helsinki.^17^ It was approved by the Swedish Ethics Authority as a multicenter study. It comprised a cohort of children identified through the centralized pediatric cardiac surgical centers in Lund and Gothenburg, Sweden. Written informed consent was given by all parents or guardians, and assent was obtained from older children and adolescents where possible. We followed the Standards for Reporting of Diagnostic Accuracy (STARD) report guideline and registered the study via the Clinical Trials website (NCT04667455). Eligibility criteria and data collection items were determined prior to commencement.

### Enrollment and Clinical Background of CHD Cases and Controls

We defined high-risk CHD in this study as cases in medical records that required cardiac surgery in infancy due to circulation dependent on patency of the arterial duct as well as cases with heart failure symptoms not responding to other types of medical care in which heart surgery was deemed necessary. Cardiac diagnoses were coded according to the *International Statistical Classification of Disease and Related Health Problems; Tenth Revision* and verified against electronic patient records (diagnosis codes included Q20 through Q28). Enrollment was based on a 2:1 case-to-control ratio. As there were no published data on the analyzed biomarkers using DBS to make accurate power calculations, we sought to minimize false-positive results among controls and false-negative results among cases through an estimation based on clinical expectations, and assumed that for the test to be applicable in clinical practice there should be 5% or fewer controls and 85% or greater cases with abnormal combined biomarker levels. To achieve 80% power with α = .05, we calculated the number of cases (175 participants) and controls (88 participants) for a total of 263.^18,19^ We identified eligible cases through electronic record queries at participating pediatric cardiac surgical centers based on diagnosis codes and age (under 18 years) while confirming diagnoses through review of surgical notes and echocardiography. We approached cases for participation chronologically, according to date of birth of the child, starting with the youngest patients first. Controls were seen postnatally either through maternity services and these were followed up for up to 1 year of age to ensure no late presentations of high-risk CHD occurred. Alternatively, we recruited controls through outpatient clinics at participating centers, during which normal echocardiographic findings were confirmed. DBS samples were retrieved in batches from the centralized Swedish national biobank for DBS samples of newborns, where these had been stored at 4 °C in an air-conditioned environment designed for long-term storage.

#### Tests for NT-proBNP and IL-1 RL1

We developed fully automated DBS immunoassays for NT-proBNP and IL-1 RL1 measurements. We coated microtitration strips with 96-well format (ThermoFisher Scientific) with either monoclonal anti–NT-proBNP antibody (HyTest Ltd) or anti–IL-1 RL1 antibody (Medix Biochemica) and labeled monoclonal tracer antibodies (HyTest Ltd for NT-proBNP; Medix Biochemica for IL-1 RL1) with Europium chelate (Eu). Excess Eu was incubated with antibody overnight, and Eu-labeled antibody preparation purified using chromatography systems with separation columns (GE Healthcare). The final product was stabilized with diethylenetriaminepentaacetic acid-bis (stearylamide) and filtered through 0.22-μm sterile syringe filters (Millipore). To prepare the DBS calibrators, we mixed artificial serum (0.9% [weight / volume] NaCl solution supplemented with a 350-μM sucrose solution) into washed red blood cells (Diaserve Laboratories). The hematocrit of this solution was checked with a hematology analyzer (Beckman Coulter). Red blood cell preparation was spiked with 6 concentrations of NT-proBNP (zero, 1500, 5000, 15 000, 50 000, and 150 000 ng/L) and IL-1 RL1 (zero, 1000, 3000, 10 000, 30 000, and 100 000 ng/L). The DBS were prepared by final dilutions of 75 μL per spot onto filter paper (Whatman). These sheets were dried overnight, then packaged and sealed in airtight foil bags with 2-g silica desiccant packets (Multisorb Technologies Inc) for storage at –20 °C until use. From the DBS samples or calibrators, 3.2-mm disks were punched (Revvity) with 3 μL of blood into wells coated previously with anti–NT-proBNP or anti-IL-1 RL1 antibody. We analyzed plates with high throughput batch analyzers (Revvity). An elution buffer volume of 150 μL per well was added to 75 ng per well tracer antibody in 5 μL and, when plated, incubated for 3 hours for NT-proBNP and 1 hour for IL-1 RL1. Next, DBS samples were removed, the plate was washed 4 times, inducer solution added (200 μL/well), and signals measured. For IL-1 RL1 sample matrix comparison we used the same assay to measure venous ethylenediamine tetraacetic acid blood levels and comparing this with DBS measurements in controls. Batched analyses were performed with laboratory staff masked to all clinical data.

### Statistical Analysis

Data were collected electronically and anonymized prior to statistical analyses using SPSS version 28 (IBM) and R version 4.2.2 (R Foundation for Statistical Computing). Results were expressed in percentages, means (SD), or medians (IQR). To allow for comparison against a standard reference test, DBS data for IL-1 RL1 were logarithmically transformed to achieve symmetric distribution.

We compared IL-1 RL1 DBS with blood plasma levels using Pearson linear correlation and Bland-Altman agreement plots. NT-proBNP DBS assay methods have been described previously.^20^ We applied logistic regression to estimate the risk outcome of the diagnostic test by modeling the log-odds of results as a linear combination of clinical and demographic variables using log10-transformed NT-proBNP and IL-1 RL1 measurements.^21^ We performed receiver operating characteristics (ROC) curve analyses to assess DBS test performances to identify high-risk CHD vs controls and considered 2-sided *P* < .05 as statistically significant.

## Results

### Congenital Heart Disease Cases and Controls

There were initially 313 participants enrolled between August 2019 and June 2023 (mean [SD] gestational age, 39.4 [1.3] weeks; 181 male [57.8%]). All participants were born between January 2005 to June 2023. There were a total of 237 cases of CHD in the sample, with 188 (79.3%) meeting our definition for high-risk CHD. Participants in the cases cohort had a mean (SD) birth weight of 3499 (486) grams and mean gestational age of 39.3 (1.3) weeks compared with the control participants, who had a mean (SD) birth weight of 3487 (479) g and mean (SD) gestational age of 39.7 (1.3) weeks (Table 1). There were no statistically significant differences between groups regarding sex, gestational age, and birth weight. The dominant lesion seen among high-risk CHD cases was coarctation of the aorta with or without transverse arch hypoplasia, followed by shunt lesions (eg, atrial or ventricular septal defects); transposition of the great arteries; single ventricles; severe valvar pulmonary or aortic stenosis; and other complex biventricular lesions such as Fallot tetralogy or heterotaxy disorders (eTable in Supplement 1).

**Table 1.** Summary of Patient Characteristics Included in Biomarker Analyses

Characteristic	Patients, No. (%)			
	Controls (n = 101)	Cases		
		High-risk CHD (n = 191)	Other CHD (n = 46)	All CHD (n = 237)
DBS samples available for analyses	96 (95.0)	188 (98.4)	29 (63.0)	217 (91.6)
Prenatal suspicion or diagnosis of CHD	NA	73 (38.8)	2 (6.9)	75 (34.6)
Normal postnatal pulse oximetry screening results	96 (100)	94 (50.0)	25 (86.2)	119 (54.8)
Discharged without CHD diagnosis after birth	NA	36 (19.1)	18 (62.1)	54 (24.9)
Sex^a^				
Female	47 (49.0)	63 (33.5)	22 (75.9)	85 (39.2)
Male	49 (51.0)	125 (66.5)	7 (24.1)	132 (60.8)
Gestational age, mean (SD) [SE], wk	39.7 (1.3) [0.1]	39.4 (1.2) [0.1]	38.8 (1.7) [0.4]	39.3 (1.3) [0.1]
Birth weight among analyzed individuals, mean (SD) [SE], g	3487 (479) [52]	3520 (481) [35]	3222 (480) [124]	3499 (486) [34]

Abbreviations: CHD, congenital heart disease; DBS, dried blood spot; NA, not applicable.

^a^ Missing or excluded patients included 5 control (4 male, 1 female), 3 high-risk CHD (3 male), 17 other CHD (10 male, 7 female), and 20 all CHD (13 male, 7 female).

### Missing Data

**Figure 1.**
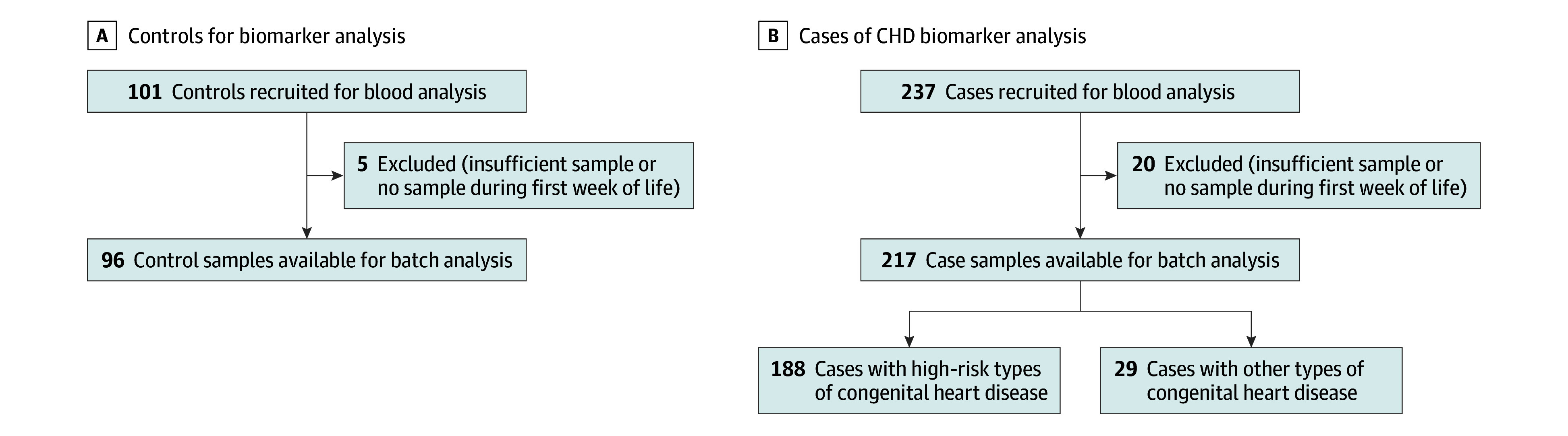
Study Flowcharts

Of 237 CHD cases, 217 could be analyzed using stored DBS samples. We had to exclude 20 cases as no or insufficient stored DBS samples were available from the national biobank. Of 101 initially enrolled controls, 5 were excluded as no DBS samples were available, leaving 96 controls for comparison with cases (Figure 1).

### Statistical Analysis for NT-proBNP and IL-1 RL1 Tests

**Figure 2.**
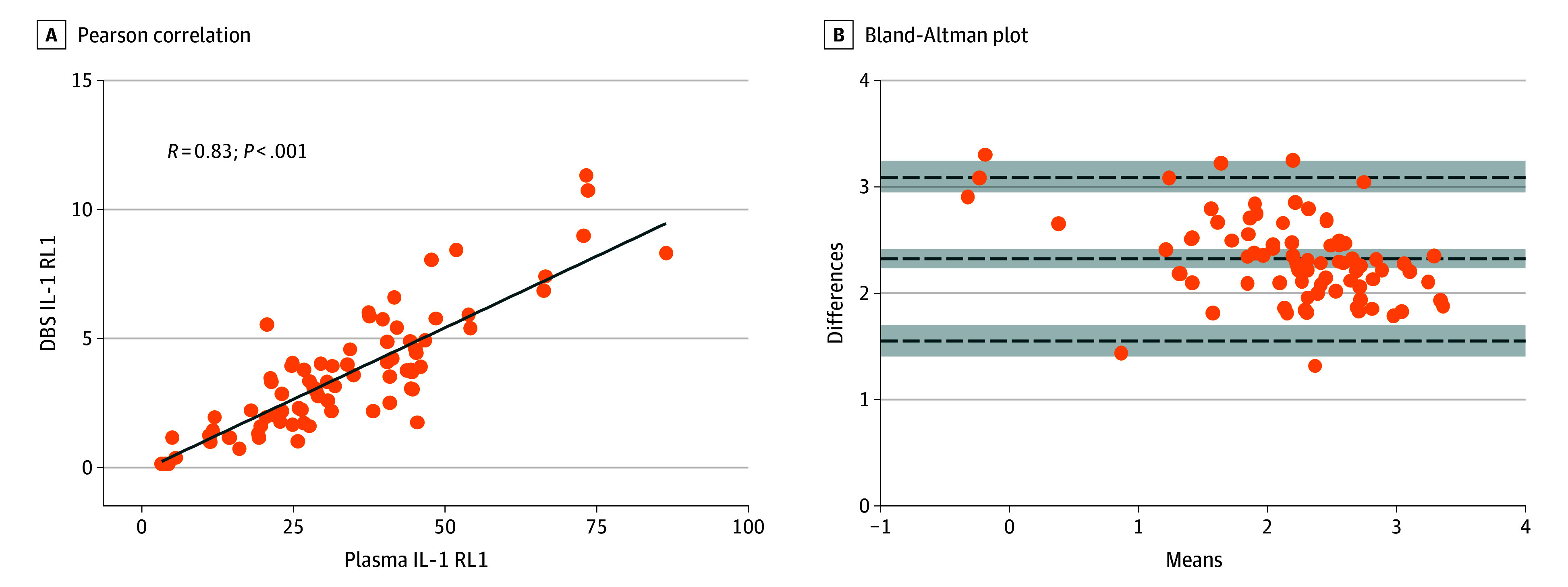
Pearson Correlation With Bland-Altman Plot of Blood Plasma and DBS IL-1 RL1 Assay Among Control Participants DBS indicates dried blood spot; IL-1 RL1, interleukin 1 receptor-like 1. Orange dots represent control cases (82 participants). In panel B, dotted lines indicate the bias (mean [SD] 2.31 [0.40]), lower limits of agreement (1.54; 95% CI, 1.39-1.69), and upper limits of agreement (3.09; 95% CI, 2.94-3.23).

DBS samples were taken at a median (IQR) 2 (2-3) days of life. Median (IQR) NT-pro-BNP levels among 217 cases was 25.5 (11.6-44.3) ng/mL and for IL-1 RL1 available levels in 215 cases showed a median (IQR) 13.4 (7.4-21.2) ng/mL. Correlation was *r* = 0.83 (*P* < .001) and test agreement showed a mean (SD) bias of 2.31 (0.40) between IL-1 RL1 DBS and blood plasma levels in 82 controls, in which both samples were taken at the same time (lower limits of agreement, 1.54; 95% CI, 1.39-1.69; upper limits of agreement, 3.09; 95% CI, 2.94-3.23) (Figure 2).

**Figure 3.**
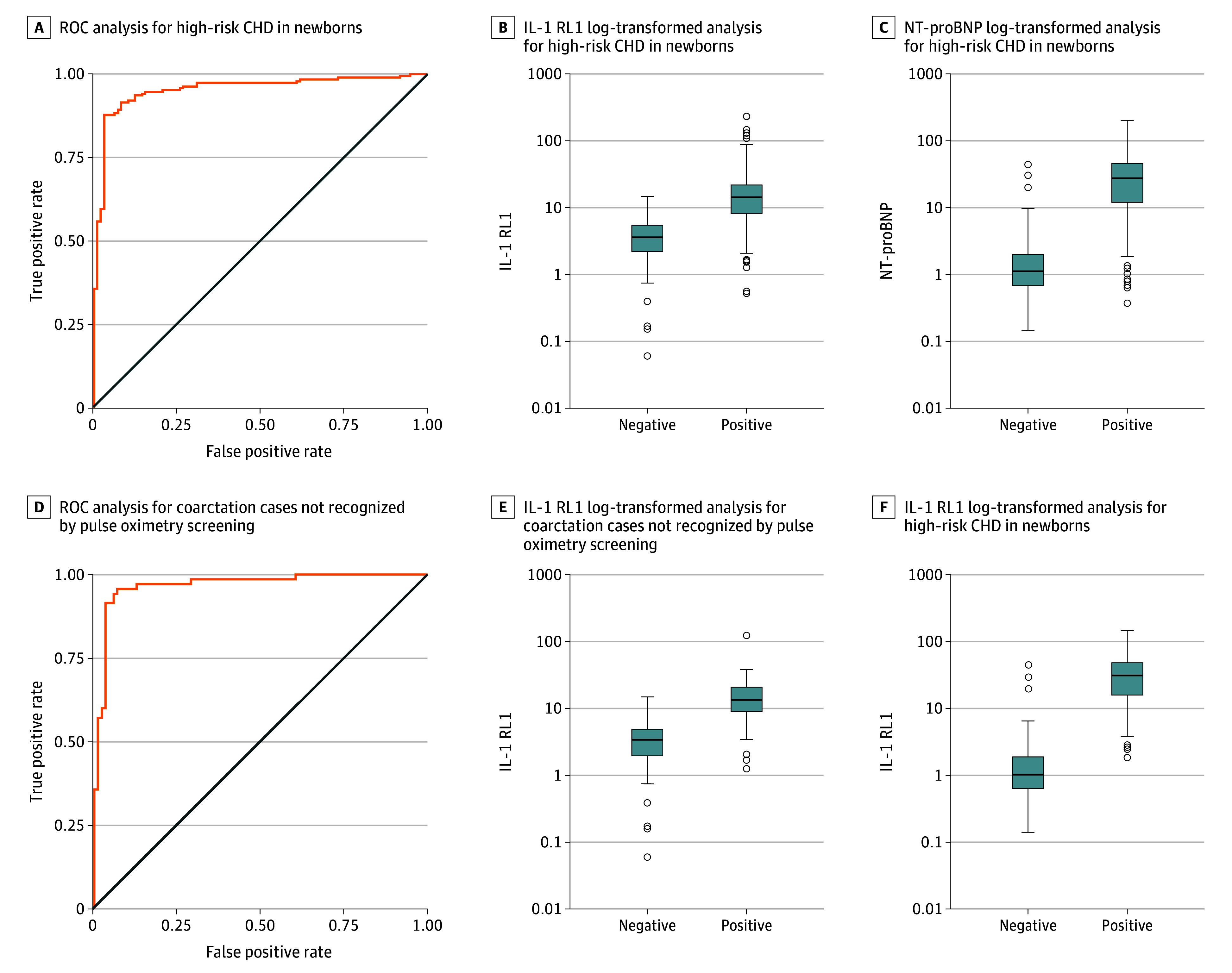
ROC Analysis for Combined NT-proBNP and IL-1 RL1 DBS Assays for High-Risk CHD and Coarctation Cases CHD indicates congenital heart disease; IL-1 RL1, interleukin 1 receptor-like 1; NT-proBNP, amino-terminal prohormone of brain natriuretic peptide; ROC, receiver operating curve. Panels A through C include 188 cases and 97 controls (AUC = 0.95; 95% CI, 0.93-0.98). Panels D through F include 70 cases and 86 controls (AUC = 0.97; 95% CI, 0.94-0.99). In panels B, C, E, and F, black bars represent the median; boxes, IQR; whiskers, 95% CIs; dots, outliers.

**Figure 4.**
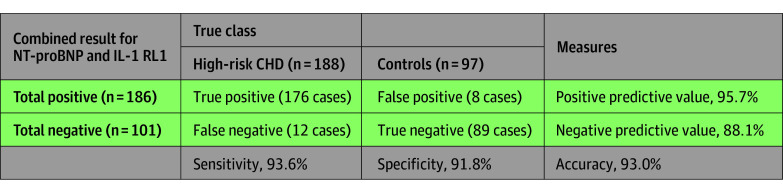
Two-by-Two Array Based on Logistic Regression for Reporting the Performance of Combined NT-proBNP and IL-1 RL1 Analyses in High-Risk CHD vs Controls CHD indicates congenital heart disease; IL-1 RL1, interleukin 1 receptor-like 1; NT-proBNP, amino-terminal prohormone of brain natriuretic peptide.

DBS assay for NT-proBNP has previously been described.^20^ Combined analysis to detect high-risk CHD in 188 cases compared with 96 controls showed a performance with AUC of 0.95 (95% CI, 0.93-0.98) (Figure 3A). For a subgroup of 70 coarctations unrecognized by postnatal pulse oximetry screening, direct comparison with 86 controls with recent results who passed universal pulse oximetry screening showed a test performance with AUC of 0.97 (95% CI, 0.94-0.99) (Figure 3B). Individual biomarker analyses for NT-proBNP showed an AUC for all high-risk cases of 0.94 (95% CI, 0.91-0.97) and for coarctations an AUC of 0.98 (95% CI, 0.95-0.99); for IL-1 RL1, all high-risk cases had an AUC of 0.90 (95% CI, 0.86-0.93) and for coarctations an AUC of 0.91 (95% CI, 0.86-0.96). Illustrations of age-related biomarker trends for sampling on days of life 2 through 4 and ROC curve for cases outside the high-risk CHD group are available in the supplement (eFigures 1 through 3 in Supplement 1). Thirty-six of 188 high-risk CHD cases (19.1%) were initially not recognized by standard prenatal or postnatal screening methods; 31 of these (86.1%) were identified as high-risk by combined NT-proBNP and IL-1 RL1 testing. By applying logistic regression to assess test performance of combined NT-proBNP and IL-1 RL1 analysis in high-risk CHD vs controls, the combined screening test showed 93.0% accuracy (sensitivity, 93.6%; specificity, 91.8%). Using this modeling, the positive predictive value of identifying high-risk CHD in this cohort was 95.7% (Figure 4).

## Discussion

### Newborns With CHD

Because of the significant global burden of CHD, and despite the advances achieved by prenatal and postnatal screening programs in many high-income countries, mortality and morbidity in infants with high-risk forms of CHD remains a challenge in many health care settings—one of the motivating factors behind this study.^4^ We excluded prematurely born babies who may have additional, milder forms of CHD, such as patency of the arterial duct, which has been assessed using standard NT-proBNP measurements.^22^ In our study, premature babies were unlikely to be discharged from neonatal care within 1 week after birth and could be clinically observed, reducing the risk of hospital discharge with unrecognized CHD. All cases were verified by experienced pediatric cardiologists through cardiac surgical notes and echocardiographic findings. Cases and controls were comparable in terms of gestational age and birth weight. Slight male predominance was seen in CHD cases, which is consistent with published reports.^23^ Using this cohort study, we successfully applied a novel approach for early diagnosis of high-risk CHD by combined DBS biomarker analysis in newborns.

### Screening Using DBS Samples

Studies to improve our knowledge about the neonatal proteome have emerged in recent years and research efforts to identify new opportunities for improvement of neonatal care using blood-based biomarkers of disease have been encouraged.^24^ We focused on novel diagnostic strategies that incorporate existing DBS newborn screening program resources into the early identification of CHD.^9,25^ To mimic routine clinical practice, we relied on DBS samples collected through the national newborn screening program in Sweden. All samples were collected during the first week of life to ensure screening for high-risk CHD fulfills general screening principles during a largely asymptomatic phase of CHD with the vast majority of DBS sampling during day 2 to 3 of life. This reflects standard clinical practice in Sweden, where routine DBS sampling is usually recommended more than 48 hours after birth. Possible test threshold limitations due to a significant drop in CHD prevalence could be overcome by maximum 2.7 test iterations to maintain a positive predictive value of 95%, which could be achieved through additional repeat blood testing in settings with lower prevalence of CHD, before referring for further echocardiographic evaluation.^26,27^ All studied samples were retrieved from cold biobank storage. We analyzed even older DBS samples, in which biomarker stability has previously been demonstrated.^28^

### NT-proBNP in Newborns With CHD

Natriuretic peptides, including NT-proBNP, have been extensively studied in humans with heart disease and are part of current national and international guidelines for the management of adults and children with heart failure.^29,30,31,32^ Elevated NT-proBNP levels have been closely linked to worse prognosis, including an increased risk for mortality, in adults with heart failure.^33,34^ NT-proBNP has been associated with cardiovascular complications in various types of CHD as well as pediatric pulmonary arterial hypertension and cardiomyopathies.^35,36,37,38,39^ As this biomarker has been established in many standard medical laboratory services, a repeat check of DBS results would appear feasible for clinical caregivers as reference ranges for NT-proBNP in children have also been published, although published data on infants is currently limited to smaller cohort studies.^12,40,41^

### IL-1 RL1 in Newborns With CHD

IL-1 RL1 belongs to the interleukin 1 family of proteins involved in intracellular signaling throughout the body such as the brain, gut, lungs, and heart.^42,43,44,45^ It has previously also been named as soluble ST2. IL-1 RL1 can be found bound to the cell membrane, although its soluble form has been most intensively studied.^46^ In its soluble form, IL-1 RL1 functions as a decoy receptor for interleukin 33 (IL-33).^47,48^ The interaction between IL-1 RL1 and IL-33 changes during increased mechanical stress within the heart, such as decompensation of congestive heart failure.^49,50^ The reduction in IL-33 by the decoy receptor function of IL-1 RL1 during such cardiac stress phases may reduce fibrosis and mediate acute inflammatory processes.^51^ The role of IL-1 RL1 in projecting the risk of adverse events in adults with heart failure appears to be independent of natriuretic peptides, with higher IL-1 RL1 levels associated with increased cardiovascular mortality based on established reference ranges.^45,52,53^ In children, this emerging cardiovascular biomarker has been studied in small cohorts with similar associations of increased IL-1 RL1 levels in patients with worsening heart function.^14,54,55^ To our knowledge, no previous study has addressed its role in neonates with high-risk CHD and no quantitative assay to measure IL-1 RL1 in DBS samples has been reported. This study emphasizes the emerging importance of IL-1 RL1 as a novel biomarker in newborns with CHD.

In this study, the combination of the NT-proBNP and IL-1 RL1 led to improvements of neonatal identification of high-risk CHD. This highlights the need to focus on novel approaches to detect potentially life-threatening CHD during infancy, while making use of established DBS newborn screening methods commonly available in many parts of the world. This would allow pediatric cardiac programs in other settings, for example, to efficiently detect infants at highest risk of collapse after birth due to CHD. To overcome centralized DBS screening program challenges of time from blood sampling to test results availability and to test whether DBS analysis in the first 24 hours of life may particularly improve identification of high-risk CHD, assessments using point-of-care assays of the studied biomarker assays should ideally be performed in prospective, population-wide research settings.

### Limitations

This study had several limitations. This was a retrospective study comprising mainly high-risk CHD cases from the current population receiving surgery in Sweden, where CHD birth prevalence has remained stable and comparable with previous published epidemiological data on CHD.^56^ Controls could not be matched individually to cases (albeit gestational age, sex, and birth weights were similar). Access to DBS samples was limited due to the study design relying on stored biobank samples. In some cases, requested material was unavailable or insufficient. We visually inspected DBS but did not formally test for possible variations in blood volumes, which may have influenced our results; just as different DBS sampling methods might in other settings.^57,58^ Subgroup analyses of specific CHD types was limited to cases with coarctation of the aorta due to small numbers of other types of congenital heart disease included in this study, such as single ventricle lesions. We attempted to minimize selection bias by approaching eligible cases according to date of birth, starting with participants born most recently. We did not record noncardiac diagnosis due to limitations of verifying diagnoses purely based on queried electronic databases nor did we analyze perinatal data, such as the type of delivery or Apgar scores, as DBS samples were routinely obtained more than 2 days after birth in our setting. We assumed that the measured biomarker levels would not be majorly affected by the initial birth and clinical status directly afterwards. As the studied DBS biomarkers may vary during the first week of life, and different references may be applicable to prematurely born babies, further assay validation should be performed before possible clinical application, especially to evaluate the emerging importance of IL-1 RL1 in this setting. This limits the generalizability of our findings and may warrant further research using prospective study design, ie, in a population-wide health care setting.

## Conclusions

This diagnostic study of neonates from Sweden proves the feasibility of analyzing NT-proBNP and IL-1 RL1 with routinely used DBS samples. Tests were accurate and performed well in differentiating healthy controls from high-risk CHD cases. This warrants prospective evaluation to improve early diagnosis of CHD in this vulnerable population of newborns.

## Notes

Supplement 1. **eTable.** Types of Congenital Heart Disease Cases Enrolled **eFigure 1.** Box Plot Diagram Illustrating Trends of Blood Biomarkers by Age **eFigure 2.** Blood Biomarker Levels Compared With Newborns With Other Congenital Heart Diseases Not Defined as High Risk **eFigure 3.** ROC Analysis for Combined NT-proBNP and IL-1 RL1 DBS Assays in Subgroup of Other Types of CHD Cases Not Considered High-Risk Compared With Controls

Supplement 2.
Data Sharing Statement
