# Supplementary material for: Newborn Screening for High-Risk Congenital Heart Disease by Dried Blood Spot Biomarker Analysis
Source: JAMA Netw Open. 2024 Jun 24;7(6):e2418097. doi: 10.1001/jamanetworkopen.2024.18097 (PMC11197454; doi:10.1001/jamanetworkopen.2024.18097)
Supplement: Supplement 1. — eTable. Types of Congenital Heart Disease Cases Enrolled eFigure 1. Box Plot Diagram Illustrating Trends of Blood Biomarkers by Age eFigure 2. Blood Biomarker Levels Compared With Newborns With Other Congenital Heart Diseases Not Defined as High Risk eFigure 3. ROC Analysis for Combined NT-proBNP and IL-1 RL1 DBS Assays in Subgroup of Other Types of CHD Cases Not Considered High-Risk Compared With Controls [file jamanetwopen-e2418097-s001.pdf]

# Supplemental Online Content

Clausen H, Friberg E, Lannering K, et al. Newborn screening for high-risk congenital heart disease by dried blood spot biomarker analysis. *JAMA Netw Open.* 2024;7(6):e2418097. doi:10.1001/jamanetworkopen.2024.18097

**eTable.** Types of Congenital Heart Disease Cases Enrolled

**eFigure 1.** Box Plot Diagram Illustrating Trends of Blood Biomarkers by Age

**eFigure 2.** Blood Biomarker Levels Compared With Newborns With Other Congenital Heart Diseases Not Defined as High Risk

**eFigure 3.** ROC analysis for combined NT-proBNP and IL1RL1 DBS Assays in Subgroup of Other Types of CHD Cases Not Considered High-Risk Compared With Controls

This supplemental material has been provided by the authors to give readers additional information about their work.

© 2024 Clausen H et al. *JAMA Network Open.*

**eTable.** Types of congenital heart disease cases enrolled (n=237). All required cardiac surgical treatment / catheter intervention. Sorted by frequency and dominant CHD lesions.

| | |
|---|---|
| Aortic coarctation (CoA) +/- transverse arch hypoplasia | 92 (38.8%) |
| Atrioventricular septal defect (AVSD), ventricular septal defect (VSD), patent arterial duct (PDA), atrial septal defect (ASD) | 37 (15.6%) |
| Transposition of the great arteries (TGA) | 34 (14.3%) |
| Single ventricle lesions (SV) | 26 (11.0%) |
| Severe aortic or pulmonary valve stenosis (AS, PS) | 18 (7.6%) |
| Heterotaxy & other complex biventricular lesions | 18 (7.6%) |
| Tetralogy of Fallot (ToF) | 12 (5.1%) |
| Total | 237 (100.0 %) |

© 2024 Clausen H et al. *JAMA Network Open.*

**eFigure 1.** Box Plot Diagram Illustrating Trends of Blood Biomarkers by Age

**A.** Box-plot diagram illustrating the trend of measured IL1RL1 values (ng/ml) in all analysed subjects according to age of sampling after birth (day 2-4 of life); no statistically significant difference related to timing of sampling was observed between cases and controls.

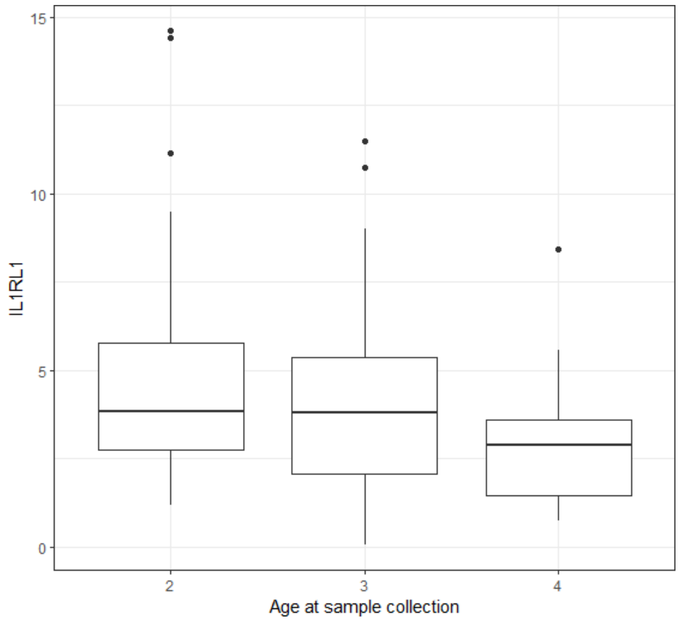


**B.** Box-plot diagram illustrating the trend of measured NT-proBNP values (ng/ml) in all analysed subjects according to age of sampling after birth (day 2-4 of life); no statistically significant difference related to timing of sampling was observed between cases and controls.

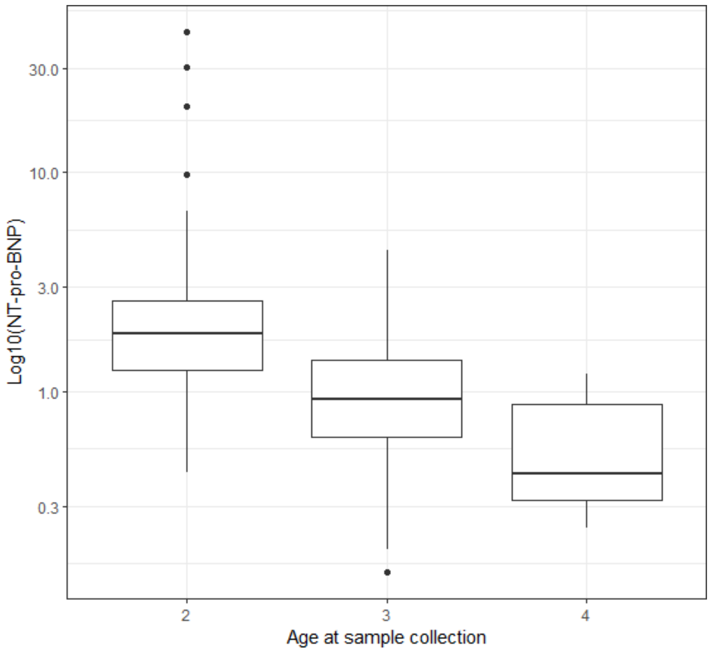


© 2024 Clausen H et al. *JAMA Network Open.*

**eFigure 2.** Blood Biomarker Levels Compared With Newborns With Other Congenital Heart Diseases Not Defined as High Risk

**A.** Box-plot diagram of IL1RL1 levels (log10-transformed data) in controls

(negative to the left) compared to other CHD not defined as high-risk cases (positive to the right).

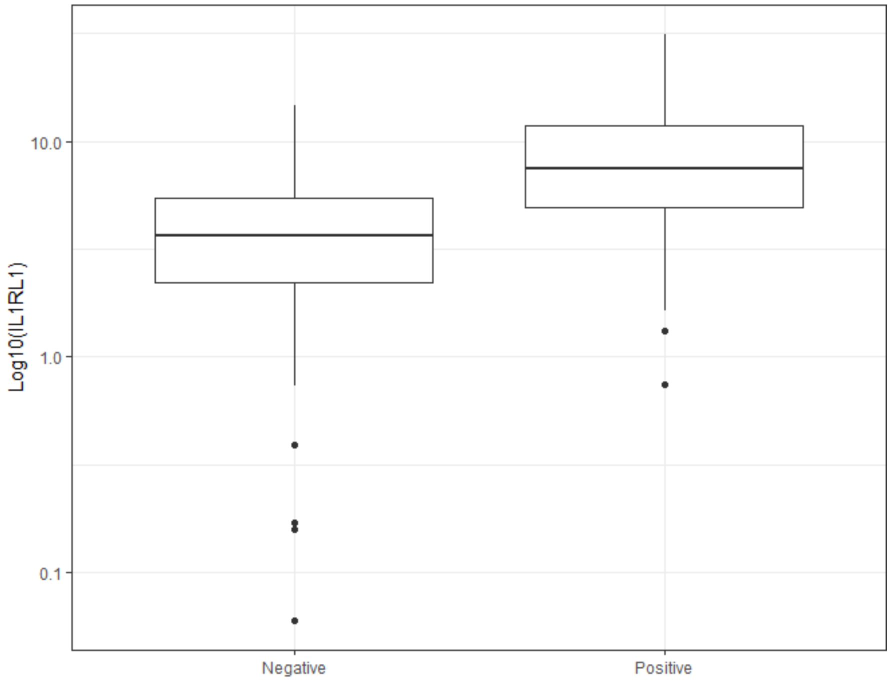


© 2024 Clausen H et al. *JAMA Network Open.*

**B.** Box-plot diagram of NT-proBNP levels (log10-transformed data) in controls (negative to the left) compared to other CHD not defined as high-risk cases (positive to the right).

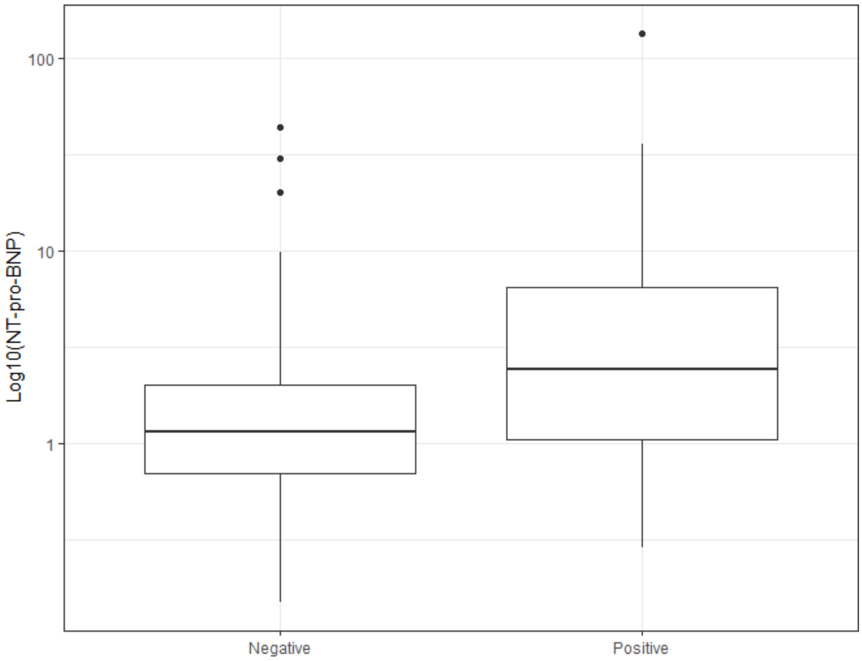


© 2024 Clausen H et al. *JAMA Network Open.*

**eFigure 3.** ROC analysis for combined NT-proBNP and IL1RL1 DBS Assays in Subgroup of Other Types of CHD Cases Not Considered High-Risk Compared With Controls

ROC analysis for combined NT-proBNP and IL1RL1 DBS assays in subgroup of other types of CHD cases not considered high-risk (n=29) compared to controls (n=86); area under curve (AUC) = 0.76 (0.63-0.88). TPR = true positive rate, FPR = false positive rate.

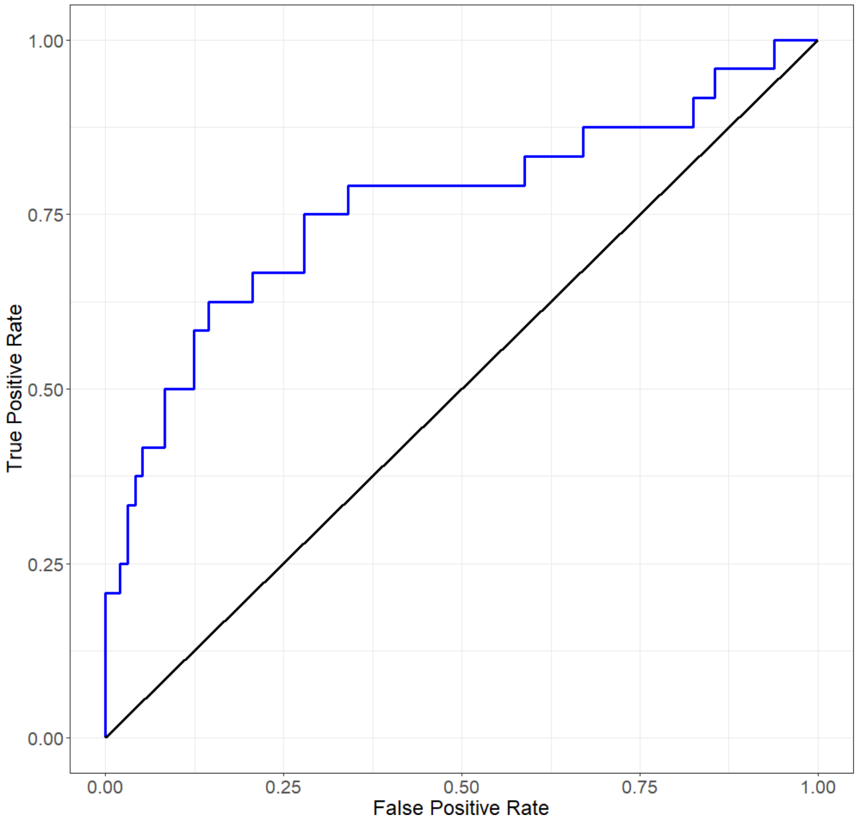


© 2024 Clausen H et al. *JAMA Network Open.*
