# Supplementary material for: Newborn Screening for High-Risk Congenital Heart Disease by Dried Blood Spot Biomarker Analysis
Source: JAMA Netw Open. 2024 Jun 24;7(6):e2418097. doi: 10.1001/jamanetworkopen.2024.18097 (PMC11197454; doi:10.1001/jamanetworkopen.2024.18097)
Supplement: Supplement 2. — Data Sharing Statement [file jamanetwopen-e2418097-s002.pdf]

Data Sharing Statement

Clausen. Newborn Screening for High-Risk Congenital Heart Disease by Dried Blood Spot Biomarker Analysis. *JAMA Netw Open*. Published June 24, 2024.
doi:10.1001/jamanetworkopen.2024.18097

Data
Data available: Yes
Data types: Other (please specify)
Additional Information: Data request can be made to the authors from non-profit / academic institutions and will be released after approval from the authors as deidentified data for research purposes only.
How to access data: Data request can be made to the authors from non-profit / academic institutions and will be released after approval from the authors as deidentified data for research purposes only.
When available: With publication

Supporting Documents
Document types: None

Additional Information
Who can access the data: Data request can be made to the authors from non-profit / academic institutions and will be released after approval from the authors as deidentified data for research purposes only.
Types of analyses: De-identified data will be made available for non-profit / academic research purposes after review of requests by the authors.
Mechanisms of data availability: After data request and review by the authors, de-identified data may be made available with signed data access agreement that will be provided by the authors in such cases.
